# Warming Up for Basketball: Comparing Traditional vs. Small-Sided Game Approaches in Youth Players

**DOI:** 10.3390/sports13120452

**Published:** 2025-12-15

**Authors:** Pierpaolo Sansone, Massimiliano Vanacore, Jorge Lorenzo-Calvo, Álvaro Bustamante-Sánchez, Alejandro Vaquera, Daniele Conte

**Affiliations:** 1 Department of Education and Sport Sciences, Pegaso Telematic University, Centro Direzionale Isola F2, 80143 Naples, Italy; pierpaolo.sansone@unipegaso.it; 2 Department of Movement Human and Health Sciences, University of Rome “Foro Italico”, 00135 Rome, Italy; massimiliano.vanacore00@gmail.com; 3 Deporte y Entrenamiento Research Group, Facultad de Ciencias de la Actividad Física y del Deporte, Universidad Politécnica de Madrid, 28040 Madrid, Spain; jorge.lorenzo@upm.es; 4 Department of Sports Sciences, Faculty of Medicine, Health and Sports, Universidad Europea de Madrid, C/Tajo, s/n, Villaviciosa de Odón, 28670 Madrid, Spain; alvaro.bustamante@universidadeuropea.es; 5 VALFIS Research Group, Institute of Biomedicine (IBIOMED), Faculty of Sciences of Physical Activity and Sports, University of León, 24007 León, Spain; avaqj@unileon.es; 6 Department of Coaching Science, Lithuanian Sports University, 44221 Kaunas, Lithuania

**Keywords:** basketball performance, internal load, external load, youth athletes

## Abstract

This study compared the external [movement load (ML)] and internal [rating of perceived exertion (RPE), mean and peak heart rate (HRmean, HRpeak)] loads, performance and enjoyment between time-matched (~12 min) traditional (TRAD) and small-sided game (SSG) warm-ups in youth basketball players. Using a counterbalanced crossover design, 24 male players (16.0 ± 0.1 years) performed both warm-up types after reporting fatigue (ROF) and completing an 8 min standardized pre-warm-up. Before and after each warm-up, players completed 20 m sprint and countermovement jump (CMJ) tests; enjoyment (ENJ) was assessed post-warm-up. No significant differences were found between warm-ups for ROF (*p* = 0.053), RPE (*p* = 0.259), or HRmean (*p* = 0.053). However, SSG induced a higher HRpeak than TRAD (*p* = 0.001), while ML was greater in TRAD (*p* < 0.001). No interaction, time effect, or typology effect emerged for performance in sprinting and change of direction, although CMJ was higher after TRAD (*p* = 0.047). Enjoyment did not differ significantly (*p* = 0.066), although with a large effect size (r = 0.612). The greater ML in TRAD may reflect more dynamic basketball actions compared with SSG, which emphasized static tasks (e.g., screening, boxing out) yet produced higher HRpeak. Coaches may consider SSG warm-ups to replicate game-specific conditions while controlling the external load and maintaining adequate physiological preparation.

## 1. Introduction

Basketball is a high-intensity, stochastic, court-based team sport with considerable physical and physiological demands [1]. During competition, basketball players are required to perform a variety of multiplanar movements, including sprints, jumps, decelerations, and static exertions, while also scanning the dynamic technical–tactical scenario and controlling the ball [1,2,3]. It is therefore evident that the demands of basketball games posit significant physical, physiological, technical–tactical and perceptual–cognitive demands on players.

In order to appropriately prepare basketball players for training and competition, warm-up procedures are an essential step [4,5]. Warm-up activities encompass a variety of training modalities, including general and specific mobility drills, injury prevention exercises, neuromuscular activation and strength, basketball-specific actions such as jumping, shuffling and changes of direction, technical actions such as dribbling, passing and shooting, and reactive, open-skill drills [4]. The progressive implementation of these varied exercises aims at closely replicating the diversified demands of the subsequent performance environment (i.e., training and games), optimizing players’ readiness and minimizing injury risk [6,7]. These benefits are obtained through a variety of mechanisms, including increased core temperature, improved oxygen uptake kinetics, post-activation performance enhancement and cognitive priming [6,8].

Previous basketball studies have described the effectiveness of warm-up strategies on subsequent player’s performance, showing that the inclusion of mobility, strength, plyometrics and reactive agility exercise can improve the players’ jumping and sprinting capacities [9,10]. While basketball literature is abundant on physical-oriented warm-up procedures, less is known about the effects of more sport-specific warm-ups. In fact, basketball games are a dynamic, open-skill environment in which players’ performance is not solely determined by physical qualities. Basketball games require players to perform around 200 ball possessions requiring continuous assessment of the technical–tactical scenarios, decision-making and response inhibition to optimally perform and avoid errors [2,11]. In fact, a recent survey on basketball warm-up practices evidenced how basketball-specific drills are the most used activities in pre-game warm-ups at national and international competitive levels [4]. Among the available training modalities, small-sided games (SSGs) might be a quite valuable choice for basketball warm-up. SSGs are typically implemented because they closely replicate the competitive scenarios from several perspectives: physical, physiological, technical–tactical and cognitive [12]. During SSGs, team sport players are immersed in an environment that is closely resemblant of the real games, therefore requiring them to perform specific movements at high intensity while also performing technical–tactical actions which are characteristic of the sport. While basketball studies have described the demands of different SSGs, demonstrating their high relevance and specificity [12,13], little is known on the effectiveness of SSGs as a warm-up procedure to appropriately prepare basketball players to compete. While previous studies have shown that SSGs induce improvements in jumping, sprinting and change of direction in soccer [14,15] and handball [16] players, which suggest their efficacy as a warm-up strategy, to the best of the author’s knowledge no study has examined SSG warm-ups in basketball settings. Therefore, the aim of this study was to compare the effects of traditional (TRAD) and small-sided game (SSG) warm-up protocols on external and internal loads, physical performance and enjoyment levels in youth basketball players.

## 2. Materials and Methods

### 2.1. Participants

Twenty-four male basketball players from two under-17 teams (twelve per team) competing in the top-tier Italian Basketball Federation (FIP) championship took part in the study (age: 16.0 ± 0.1 years; stature: 1.82 ± 0.07 m; body mass: 73.8 ± 9.1 kg; playing experience: 8.6 ± 3.1 years). An a priori power analysis determined a minimum sample size of 11 players (α = 0.05, β = 0.95, effect size = 1.23), based on previous research comparing warm-up protocols in elite handball players [16]. All players were free from injuries affecting performance in the month before testing. The study was approved by the University of Rome “Foro Italico” ethics committee (CAR. 192/2024).

### 2.2. Design

This study encompassed a randomized cross-over design, in which participants performed physical tests before and after two different warm-up typologies: traditional (TRAD) and small-sided games (SSG). The full experimental protocol was carried out one to two weeks after the completion of the in-season period. The 24 players were divided into groups of 6 based on coaching staff’s suggestions to create balanced teams for the SSGs. Each group was composed of four guards, as well as small forward (positions 1-2 and 3) and two power forward and/or center (positions 4-5). The two protocols were completed on two separate testing days, approximately one week apart. The order of warm-up strategies was randomized and counterbalanced. Furthermore, a familiarization session was conducted during the preceding week, in which players were introduced to the testing procedures, rating scales, and both warm-up protocols.

**Figure 1:**
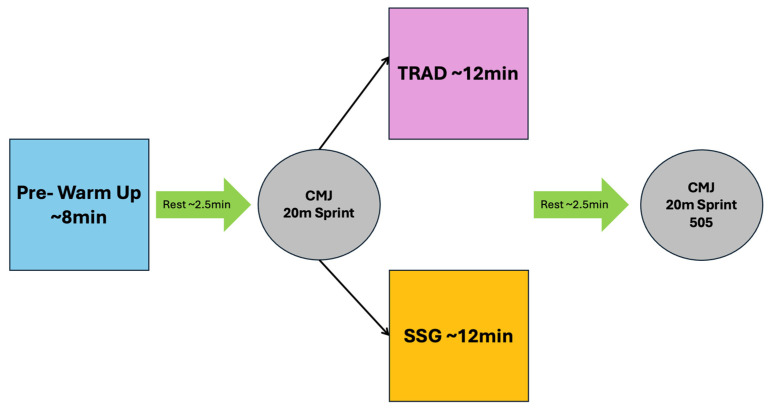
Study design. Note: TRAD = traditional warm-up; SSG = small-sided game warm-up; CMJ = Countermovement Jump Test; 505 = 505 test.

The structure of each experimental session (TRAD and SSG) is displayed in Figure 1. The experimental sessions were preceded by an 8 min introductory warm-up, performed without the use of the ball and supervised by a strength and conditioning coach accredited by the Italian Basketball Federation. Following this, after ~2.5 min of recovery, players completed countermovement jump (CMJ) and 20 m sprint tests, which served as baseline values. They then performed one of the two warm-up protocols, each lasting approximately 12 min. At the conclusion of these protocols, and after ~2.5 min of recovery, players were re-assessed using the CMJ test, the 20 m sprint test, and the change of direction test (5-0-5). The recovery period of 2.5 min was selected based on a detailed analysis of basketball competition, which demonstrated that this interval typically corresponds to the time between the conclusion of the warm-up and the commencement of official matches.

### 2.3. Procedures

#### 2.3.1. Warm-Up Typologies

The pre-warm-up was structured to follow a progressive sequence of movements, commencing with ground-based exercises and subsequently advancing to upright activities (Supplementary Tables S1 and S2). The proposed exercises were designed in accordance with evidence-based recommendations for warm-up protocols reported in the literature [6,17,18]. The session was divided into two primary components: Pillar Preparation, including as a series of exercises aimed at enhancing joint mobility and stability of the major kinetic segments, and Movement Preparation, consisting of dynamic stretching drills and sport-specific locomotor patterns. Collectively, these exercises were intended to elicit neuromuscular readiness and to prepare athletes for acceleration, jumping, and change-of-direction demands typically observed during competitive play or, in this case, during motor performance testing.

**Figure 2:**
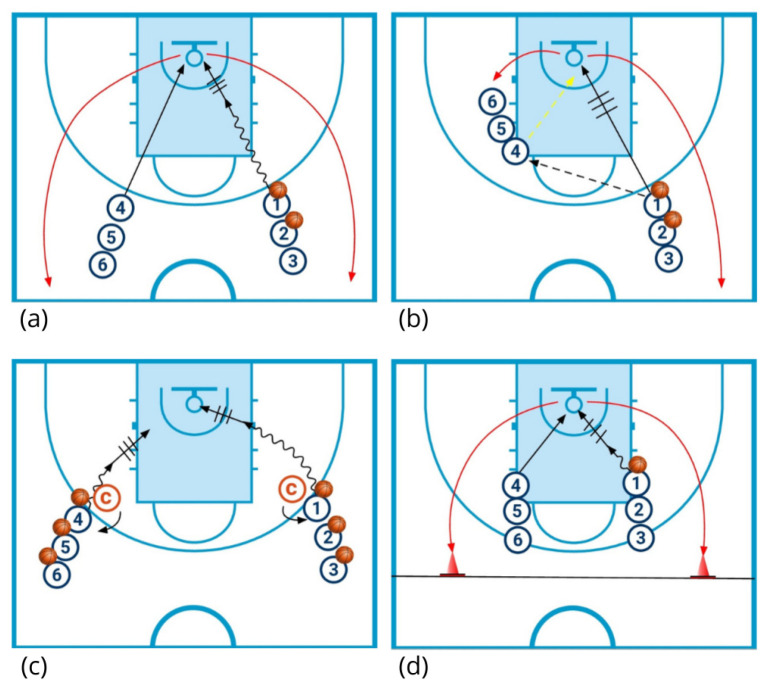
Exercises performed during the traditional warm-up: (**a**) lay-up drill; (**b**) “give-and-go” drill; (**c**) dribble and shot decision drill; (**d**) fast lay-up drill without dribbling. Symbols 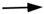: off-ball run; 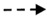: ball pass; 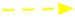: give-and-go pass; 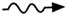: movements while dribbling the ball; 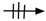: shooting; 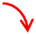: switch lines; 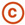: coach; 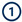 players without ball; 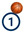 players with ball.

The traditional warm-up (Figure 2) was designed to reflect the activity commonly performed by youth basketball teams during pre-game routines. It was performed on a half-court by one of the two groups of six players and consisted of four drills:

(1) Lay-up drill: Players are divided into two lines at the wing positions, with three players on the right wing and three on the left wing. The first player in the right-hand line begins by dribbling the ball and finishing with a layup. Simultaneously, the corresponding player from the left line collects the rebound, passes the ball to the next player in the right-hand line, and joins that line. The shooter rotates to the opposite line. The drill lasts a total of 4 min, 1 min and 30 s per side, with 30 s allocated for switching sides.

(2) Give-and-go drill: Three players line up at the right-wing position and three at the left elbow. The first player on the right wing passes the ball to the first player at the left elbow and then cuts toward the basket. The cutter receives a return pass and completes the action with either a layup or a mid-range jump shot. The passer at the elbow grabs the rebound and joins the right-wing line after passing the ball to the next player in that line. The shooter rotates to the elbow line. The drill lasts three minutes in total, with approximately 1 min and 20 s per side and 20 s for switching sides.

(3) Dribble and shot decision drill: Each player has a ball and positions themselves at the extended elbow, with three players per side. Two staff members act as passive defenders, standing in front of the first player in each line. Players initiate their move based on the space left open by the staff member, selecting the appropriate finishing option. If the area near the basket is occupied, the player executes a mid-range shot; if it is free, the player drives to the basket and finishes with a layup or another solution. The drill lasts 3 min in total, divided into 1 min and 20 s per side, plus time for switching lines.

(4) Fast lay-up drill without dribbling: The initial setup of the first drill is reused for this final drill. In this variation, players are not permitted to dribble before executing the layup, making it a faster-paced activity typically performed immediately before tip-off. The rebounder passes the ball to the next player for the layup, then sprints to the sideline near the bench area before returning to perform another layup. This drill lasts two minutes in total, divided into one minute per side.

**Figure 3:**
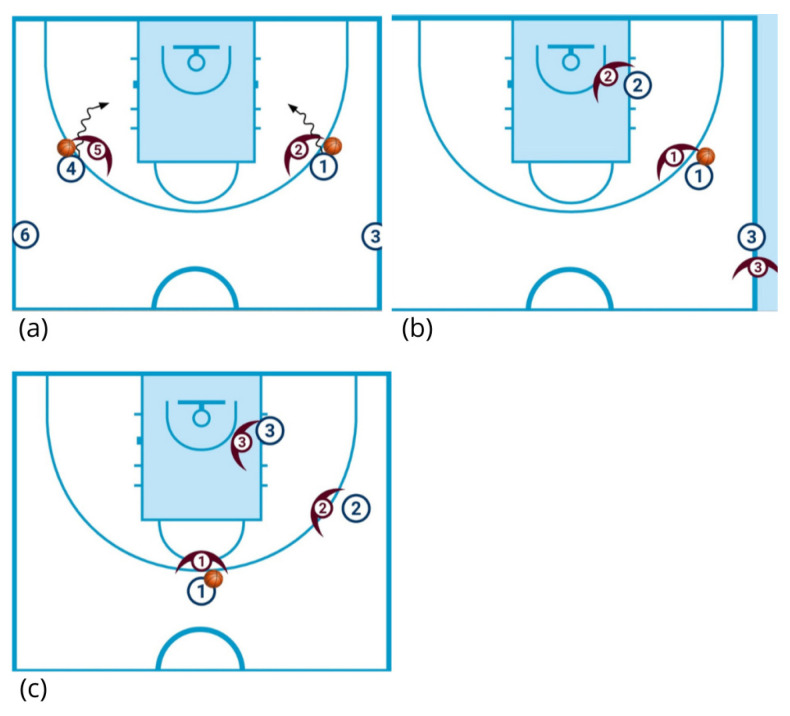
Exercises included in the SSG (small-sided game) warm-up: (**a**) 1v1; (**b**) 2v2; (**c**) 3v3. Symbols 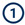: offensive player without ball; 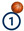 offensive player with ball. 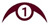: defensive player; 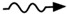: movements while dribbling the ball.

The SSG warm-up (Figure 3) was structured to provide a situational progression, moving from 1v1 to 3v3. This warm-up was performed on a half-court by one of the two groups of six players, simultaneously with the TRAD warm-up conducted on the opposite half-court. It consisted of three drills with a total duration of approximately twelve minutes:

(1) 1v1: The six players are divided into two groups of three on each wing. The first pairs on each side begin playing 1v1 from the wing position simultaneously. After the initial possession, the offensive player becomes the defender, while the waiting player (the third in line) rotates in as the new offensive player. The defender from the first possession rests until the second possession concludes, at which point they become the offensive player in the subsequent sequence. Two constraints were imposed on the offense: (1) ball handling was limited to a maximum of four dribbles to regulate the duration of each 1v1 action, and (2) players were forbidden from crossing the imaginary midline extending from the basket to midcourt. This rotation continues for two minutes per side before switching wings for an additional two minutes.

(2) 2v2: Players are paired according to playing positions, forming three balanced pairs by role. The first team begins on offense against the second team, with the offensive setup consisting of a ball-handler at the wing and a teammate positioned at the low post. Following the first possession, the defensive team rests until the conclusion of the next possession, while the third team (waiting during the first possession) becomes the offensive team in the second sequence. The initial offensive team switches to defense in the second possession. This offense–defense rotation continues for four minutes, with the low-post position alternating sides whenever teams return on offense. Players were encouraged to incorporate pick-and-roll actions during this SSG drill and were free to include any type of defense.

(3) 3v3: The six players are divided into two teams of three, competing in 3v3 with offense and defense alternating after each possession. Play begins with the offensive players positioned at the top (point guard), wing, and low post on the same side. In the following offensive sequence, the alignment shifts to the opposite wing and low-post side. If no basket is scored, play continues through both offensive and defensive rebounding. The drill lasts a total of five minutes, consisting of two minutes of play and one minute of passive rest, followed by another two minutes of play. After each scored basket, teams are allowed a five-second recovery, included within the total drill duration.

#### 2.3.2. Load Monitoring

Each warm-up protocol was monitored using both external and internal load measures. For the assessment of external load, Firstbeat Sport Sensors (Firstbeat, Yliothispistonkatu, Finland) equipped with a triaxial accelerometer sampling at 50 Hz were employed. Specifically, values for Movement Load and Movement Intensity (both expressed in arbitrary units [A.U.]) were calculated over the duration of each warm-up protocol (TRAD and SSG). Movement Load was derived using a previously described formula, which accounts for accelerations across the three axes [19]. Movement Intensity was defined as the mean Movement Load per minute.

For the evaluation of internal load, the same devices were used with a sampling frequency of 1 s. The collected data were subsequently downloaded and processed via the proprietary software. Objective measures of internal load included average heart rate (average HR) and peak heart rate (peak HR), as recorded by the Firstbeat system. In addition, subjective internal load was assessed using the Rating of Perceived Exertion (RPE), based on the modified CR10 scale proposed by Foster et al. (2001) [20]. RPE data were collected at the conclusion of each warm-up protocol, with participants individually reporting their values in the presence of the research team, ensuring that responses were provided without influence from other athletes.

#### 2.3.3. Performance Tests

The CMJ test with arm swing was used to assess jumping performance through the Optojump system (Optojump, Microgate, Bolzano, Italy). Players started in the erect standing position (with feet placed hip-width to shoulder-width apart) and were instructed to jump “as high and as fast as possible” [21] using a self-selected countermovement depth. Players performed two trials, separated by ~2 min of passive rest, with the highest jump achieved at each time point being used for analysis [21]. Excellent test–retest reliability has been previously shown for this procedure [22]. Furthermore, maximal sprint performance over 10 m and 20 m was assessed using a 20 m sprint test with a 10 m split using three sets of timing gates (Racetime 2 Light Radio Kit, Microgate, Bolzano, Italy) positioned 0, 10, and 20 m from the start line [23]. Moreover, change of direction (COD) ability was assessed using the 505 test employing the same timing gate system described above positioned 10 m from the starting line, with cones placed at the 15 m mark [24]. Participants sprinted from the start, passed through the timing gates, continued to the cones, executed a 180° turn, and then sprinted back through the timing gates [24]. The time taken to cover the distance from the 10 m gate to the 15 m cones and back to the 10 m gate was recorded as the 505 COD performance.

#### 2.3.4. Perceived Fatigue and Enjoyment

The overall perception of fatigue was asked to each player before each experimental session using the previously validated Rating-of-Fatigue (ROF) scale [25], ranging between 0 (“not fatigued at all”) and 10 (“total fatigue and exhaustion—nothing left”). This scale was also previously used with basketball athletes [26]. Additionally, a previously validated scale was adopted to assess players’ enjoyment levels on a 1–7 Likert scale [27] at the end of each warm-up typology. This scale was previously used in basketball athletes [28].

### 2.4. Statistical Analysis

For each dependent variable analyzed with parametric statistics, means and standard deviations were calculated as descriptive statistics. Successively, linear mixed models were employed to analyze jump (CMJ) and sprint (10 and 20 m) performance, with two independent variables: (1) warm-up type (TRAD vs. SSG) and (2) time (pre- vs. post-warm-up). Due to technical sampling issues, sprint data were available for only 12 players (one team). For the 505 test results and for external and internal workload measures (movement load, movement intensity, average HR, peak HR), linear mixed models were also applied, but including only one independent variable (warm-up type). In all models, “player” was specified as a random effect. The LMM analyses were run using the Jamovi software (version 2.3.21, retrieved from https://www.jamovi.org accessed on 28 August 2025). The t statistics calculated from the LMM were then converted into Cohen d effect sizes and associated 95% CI using the RStudio software (version 4.2.1). Effect sizes were interpreted as follows: <0.2, trivial; 0.20 to 0.59, small; 0.60 to 1.19, moderate; 1.2 to 1.99, large; and ≥2.0, very large [29].

For the perceptual measures RPE, ROF, and ENJ, differences between the two warm-up types were analyzed using the Wilcoxon signed-rank test (non-parametric analysis) and descriptive statistics were calculated with median and interquartile range. The non-parametric analysis was calculated using the Jamovi software previously described. Moreover, effect sizes for pairwise comparison were calculated using the r-value [30] and were interpreted according to Cohen’s benchmarks as no effect (0–0.09); small (0.10–0.29); medium (0.30–0.49); and large (≥0.5) [31]. For all statistical tests, the significance level was set at *p* < 0.05.

## 3. Results

**Figure 4:**
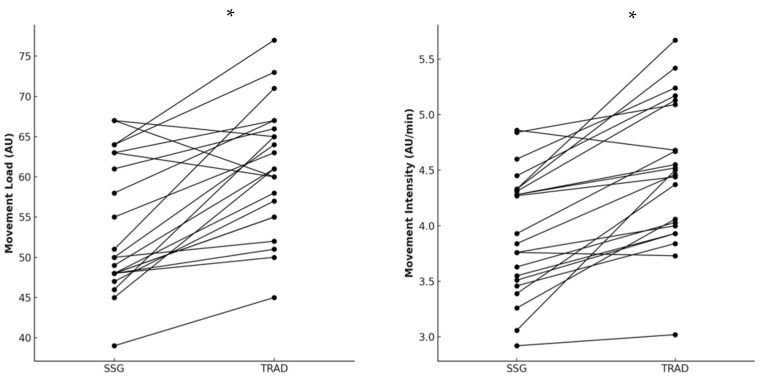
Differences in movement load and movement intensity between small-sided game (SSG) and traditional (TRAD) warm-up. Note: * indicates *p* < 0.001.

TRAD warm-up showed higher movement load [*p* < 0.001; ES(95%CI) = 1.54 (0.88; 2.20); large] and movement intensity [*p* < 0.001; ES(95%CI) = 1.80 (1.11; 2.48); large] compared to SSG warm-up (Figure 4).

**Figure 5:**
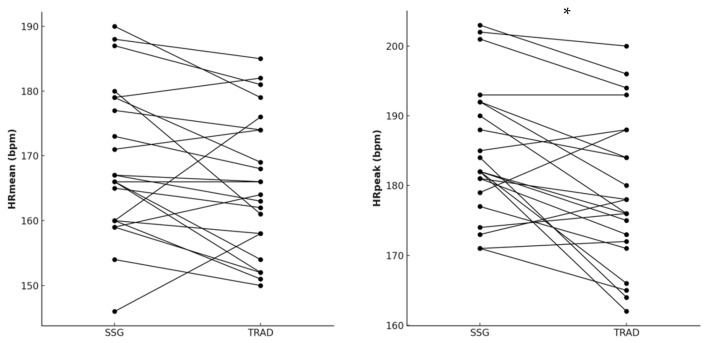
Differences in mean heart rate (HRmean) and peak heart rate (HRpeak) between small-sided game (SSG) and traditional (TRAD) warm-up. Note: * indicates *p* = 0.001.

Conversely, higher HRpeak [*p* = 0.001; ES(95%CI) = −1.07; (−1.69; −0.46); moderate] was found in SSG compared to TRAD warm-up. Moreover, moderately higher HRmean [ES(95%CI) = −0.61 (−1.20; −0.01)] was found also in SSG compared to TRAD warm-up, although with no statistical difference (*p* = 0.053) (Figure 5).

**Figure 6:**
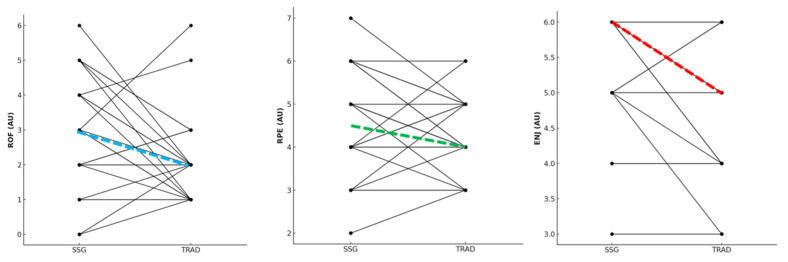
Differences in rate of fatigue (ROF), rating of perceived exertion (RPE) and enjoyment (ENJ) between small-sided game (SSG) and traditional (TRAD) warm-up. Note: dashed lines indicate the connection between the median values.

The analysis of RPE showed no significant difference between the two warm-up typologies (*p* = 0.259; r = 0.270; small) (Figure 6).

Considering performance tests, no differences were evident between conditions for each physical test, except for higher CMJ values [*p* = 0.047; ES(95%CI) = −0.58 (−1.16; −0.01), small] found in TRAD compared to SSG warm-up (Table 1).

Finally, SSG warm-up showed a moderately higher ROF (r = 0.443) and a largely (r = 0.612) higher enjoyment compared to TRAD warm-up, although both results showed non-statistically significant differences (*p* = 0.053 and *p* = 0.066 for ROF and enjoyment, respectively) (Figure 6).

## 4. Discussion

The aim of this study was to compare the demands of a traditional basketball warm-up with those of an SSG of equated duration. The main findings of this study were that (i) TRAD had higher external load and intensities, likely due to higher distances covered and more jumps performed; (ii) HR responses were higher in SSG, possibly induced by more static exertions alongside heightened psychological and cognitive demands; (iii) similar performance improvements were induced for sprinting and COD, while CMJ height was higher after TRAD, possibly because of minor variations in the athlete’s conditions across experimental days; and (iv) players expressed higher ROF after SSG than TRAD, while RPE was similar across conditions.

Between the two warm-ups, TRAD had higher external loads and intensities compared to SSGs while inducing similar performance improvements. This fact should be well considered by basketball practitioners since accumulating excessive external loads before an official game might not be desirable. This is particularly true for starting players, who are exposed to greater external loads during games and might develop fatigue earlier [32]. Differently, HRmean and HRpeak were moderately higher in SSG. This contrasting result between external and internal load outcomes is critical for basketball coaches and practitioners. It is possible that the actions performed during SSGs reflect typical basketball scenarios in which players fight for their position on the court—such as boxing out, setting picks, or post moves. These actions involve more static exertions, which are typically executed during basketball game-play (1.5 occurrences per minute) [33]. While these quasi-isometric actions do not feature positional displacement, and therefore do not impact external loads, they increase the player’s HR [1]. Since SSGs were played on half-courts and with opposition, it is plausible that players had higher HR responses under this condition because of increased static exertions, which were not featured in the TRAD warm-up. Furthermore, previous research has shown that increased cognitive demands lead to heightened HR responses [34]. During the SSGs, players likely experienced greater mental demands, induced by the greater complexity of the scenario in which they had to continuously evaluate the opponents’ and teammates’ actions, while on the other hand, the TRAD warm-up required only internally paced activities. Therefore, the higher mental demands of SSGs might have also contributed to the higher HR responses found.

One of the main goals of a warm-up is to enhance the player’s physical capacities [4]. In the current study, none of the protocols led to significant improvement in performance tests. A possible explanation is the 8 min pre-warm-up performed before each condition, which may have masked the effects of the subsequent TRAD and SSG warm-ups. While a pre-warm-up is necessary to prepare players for baseline assessments (e.g., CMJ, sprint) and is commonly included in similar studies investigating warm-up strategies (REF), it is possible that its execution had already primed the players for simple actions such as jumping and sprinting. Consequently, the additional TRAD and SSG warm-ups may not have elicited further post-activation potentiation effects. This is one of the key outcomes of this study and it should be considered also when designing future research with similar aims.

Comparing the effects of TRAD and SSG warm-ups on performance outcomes, no prominent differences were found. While a small significant difference was found across warm-ups, this might be due to minor variations in the athletes’ conditions across experimental days. This hypothesis is supported by the absence of time effects in either condition, and the higher ROF reported by players in the SSG day, while perception of effort was similar. It appears therefore that SSGs can be used as a warm-up strategy in youth basketball players as they do not differ from TRAD warm-ups in terms of physical preparation of players. Furthermore, SSGs might be preferred over TRAD as they stimulate technical–tactical and cognitive abilities while preparing players physically and physiologically [12], thus being a more effective and efficient training choice.

From the perceptual standpoint, TRAD and SSG warm-ups induced similar perceptions of effort (i.e., RPE scores), while ENJ was largely higher for SSGs. Our findings corroborate previous studies which demonstrated that the ball content of SSGs elicits positive affective responses in team sport players [35,36]. Thus, while perceived demands were similar, the SSG warm-up was preferred by players, making it an optimal choice for youth basketball coaches who want to keep their players in good spirit and well motivated.

Although this study provides important insights for basketball coaches and practitioners, some limitations should be acknowledged. Firstly, the external load was monitored only with proxy variables, while more details might have been obtained using different technologies which provide specific activity profiles (i.e., accelerations, jumps, changes of direction). Secondly, player’s readiness following the warm-up conditions was measured only by means of a physical performance test, without considering the mental and technical–tactical readiness, which are essential for team sports [37]. Additionally, the outcomes of our study are referred only to youth male players, limiting the generalizations to other basketball populations. Finally, our players were only tested on two occasions, highlighting the short-term effects of the two warm-up protocols, with no information available about the long-term responses (i.e., during the actual training sessions or official match). Therefore, future studies should (i) compare basketball warm-up procedures evaluating their activity profiles in more detail; (ii) evaluate the effects of different warm-ups on the psychological and technical–tactical readiness of players; (iii) replicate this study in other basketball populations (i.e., female and/or adult players) to better generalize the results; and (iv) include a long-term analysis to verify the effect of different warm-up strategies on the post-warm-up activities.

## 5. Conclusions

Based on the outcomes of this study, basketball coaches are encouraged to adopt SSG-based warm-ups when aiming to reduce external load while eliciting higher internal demands through game-based scenarios involving static positions (e.g., picking, screening). When preceded by a pre-warm-up routine, both SSG and traditional warm-ups can be used interchangeably to prepare players for jumping, sprinting, and change-of-direction performance. Importantly, given the substantially higher enjoyment reported during SSG compared to traditional warm-ups, coaches should consider prioritizing this modality to enhance players’ enjoyment and, potentially, their adherence to training across the season. Overall, SSG warm-ups not only can prepare players physically in the short term but also increase their enjoyment—both of which are important for optimal performance.

## Figures and Tables

**Table 1:** Mean SD for jumping and sprinting performances before and after each warm-up typology.

Dependent Variable	TRAD		SSG		P Interaction	P WU Typology	P Time
	PRE	POST	PRE	POST			
CMJ (cm)	47.5 ± 11.4	47.4 ± 11.6	46.6 ± 11.3	46.7 ± 11.6	0.804	0.047	0.996
20 m (s)	3.18 ± 0.90	2.91 ± 0.89	2.93 ± 0.89	2.93 ± 0.89	0.459	0.680	0.592
10 m (s)	1.86 ± 0.12	1.70 ± 0.51	1.72 ± 0.52	1.69 ± 0.51	0.959	0.796	0.140

Note: CMJ = Countermovement Jump; 20 m = 20 m sprint time; 10 m sprint time; TRAD = traditional warm-up; SSG = small-sided game warm-up; WU = warm-up.

## Data Availability

The original contributions presented in this study are included in the article/Supplementary Materials. Further inquiries can be directed to the corresponding author.

## Supplementary Materials

The following supporting information can be downloaded at https://www.mdpi.com/article/10.3390/sports13120452/s1: Table S1: Pillar preparation structure: type of exercise, number of repetitions to perform, and exercise goal.; Table S2: Movement preparation structure: type of exercise, number of repetitions to perform, and exercise goal.
