# Supplementary material for: Warming Up for Basketball: Comparing Traditional vs. Small-Sided Game Approaches in Youth Players
Source: Sports (Basel). 2025 Dec 15;13(12):452. doi: 10.3390/sports13120452 (PMC12737053; doi:10.3390/sports13120452)
Supplement: Supplementary file 1 [file sports-13-00452-s001.zip › sports-3925733-supplementary.pdf]

Supplementary Table S1. Pillar preparation structure: type of exercise, number of repetitions to perform, and exercise goal.

**Pillar preparation (mobility, dynamic stretching and stability)**		
Exercise	**Repetitions**	**Objective**
Hamstring floss	10 each side	Dynamic hamstring stretching
Supine hip internal/external rotation	5 each side	Hip mobility and dynamic hip flexor stretching
Supine leg lowering	6 each side	Dynamic hamstring stretching
Dead-bugs	5 each side	Anti-extension stability and core activation
Bridge	10	Core stability and glute/hamstring activation
Bridge one leg w/ hip flexion	6 each side	Glute and hamstring stability and activation
Bridge march	10	Posterior chain stability
Hip 90-90 extra-/intra-rotation	5 each side	Hip mobility
Hip dynamic 90-90	10	Hip mobility
Cat–camel	10	Spinal mobility
Quadruped scapular adduction/abduction	10	Anti-extension stability and scapular mobility
Down-dog	5	Dynamic posterior chain stretching

Supplementary Table S2. Movement preparation structure: type of exercise, number of repetitions to perform, and exercise goal.

**Movement preparation (dynamic stretching, movement integration and neural activation)**		
Exercise	**Repetitions**	**Objective**
Run forward w/ shoulder rotations	2	Increase in body temperature, scapulohumeral mobility
Lateral canter w/ arm movement	2	Increase in body temperature and scapulohumeral mobility
Knee hug		
Run	1	Dynamic hamstring stretching<br>Increase in body temperature
Quads stretch w/ arm raise	1	Dynamic quadriceps and anterior chain stretching
Run	1	Increase in body temperature
Leg cradle	1	Dynamic hip external rotator stretching
Back-pedaling	1	Increase in body temperature and quadriceps activation
Single-leg deadlift w/ reach	1	Dynamic posterior chain stretching and body stability
Back-pedaling	1	Increase in body temperature and quadriceps activation
Front lunges w/ thoracic rotations	1	Dynamic hip flexor/extensor stretching and thoracic mobility
Back lunges w/ thoracic extension	1	Dynamic hip flexor/extensor and anterior chain stretching
Lateral lunges	2	Dynamic adductor stretch and hip mobility
Pillar march	2	Dynamic stability and acceleration pattern
Pillar skip	2	Plyometric activation, rhythm, and acceleration pattern
Linear bound	2	Horizontal plyometrics and acceleration pattern
Fall acceleration w/ close-out	2	Acceleration and technical component
Lateral pillar skip	2	Anti-lateral flexion stability and shuffle pattern
Lateral bound	2	Lateral/horizontal plyometrics and shuffle pattern
Fall acceleration w/ close-out and shuffle	2	Specific neural activation
Cognitive hip-rotation shuffle and acceleration	2	Specific neural activation
